# Scatology and behavioural observations yield different estimates of the dietary composition of Western Capercaillie *Tetrao urogallus*

**DOI:** 10.1038/s41598-026-50558-3

**Published:** 2026-04-29

**Authors:** Grzegorz Orłowski, Dorota Merta, Janusz Kobielski, Jerzy Karg, Joanna Czarnecka

**Affiliations:** 1 Institute of Technology and Life Sciences, National Research Institute, Falenty, Al. Hrabska 3, Raszyn, 05-090 Poland; 2 Institute of Biology and Earth Sciences, University of the National Education Commission, 2 Podchorążych Str, Kraków, 30-084 Poland; 3 Ruszów Forest Inspectorate, Leśna 2, Ruszów, 59-950 Poland; 4 University of Zielona Góra, Góra, Poland; 5 Department of Botany, Mycology and Ecology, Institute of Biological Sciences, Maria Curie-Skłodowska University, 19 Akademicka Street, Lublin, 20-033 Poland

**Keywords:** Galliforms, Dietary habits, Plant food, Food ingestion, Food digestion, *Vaccinium* spp., Conifer needles, Fecal analysis, Animal droppings, Ecology, Ecology, Plant sciences

## Abstract

Comparative evaluations of methods used to assess animal diets remain scarce, despite their importance for ecological research and species conservation. Here, we compared diet composition of the Western Capercaillie *Tetrao urogallus*, a large forest herbivorous grouse, estimated using two commonly applied approaches: direct behavioural observations of food consumption (BEH) and microhistological analysis of faeces (scatology; MIC). Owing to limited taxonomic resolution in behavioural observations, food items were grouped into four major classes: *Vaccinium* spp., coniferous trees (predominantly Scots pine *Pinus sylvestris*), monocotyledonous taxa, and other plant material (mainly supplemental food). Diet composition differed significantly between methods. Estimates based on MIC and BEH diverged primarily in the relative contribution of *Vaccinium* plant parts and coniferous trees. The monthly mean proportion of *Vaccinium* was nine times lower in MIC than in BEH estimates (7% vs. 64%), whereas the contribution of coniferous trees was three times higher in MIC than in BEH (71% vs. 24%). Within faecal samples, remains of *Vaccinium* seeds were disproportionately more frequent than leaves (66% vs. 32%), indicating greater degradation of leaf tissues and suggesting preferential consumption of berries over leaves, thereby providing evidence of frugivory. Despite these substantial differences in absolute percentages, both approaches revealed broadly similar seasonal patterns for the dominant food types. These differences likely reflect variation in temporal integration (daytime foraging vs. cumulative digestion, including feeding at roost sites) and differential post-digestive preservation of plant tissues, rather than methodological inaccuracy. Our results indicate that BEH and MIC should not be interpreted as interchangeable estimates of diet composition but rather as complementary indicators reflecting different temporal and behavioural aspects of food intake.

## Introduction

Understanding the dietary ecology of animals is essential for gaining insight into their habitat requirements, trophic interactions and food webs^1^. Dietary studies are crucial for conservation as they reveal how food choices affect an animal’s environment and surrounding biodiversity^2,3^. For reintroduction projects, when the species has been absent for a prolonged period, understanding the complex interplay between habitat and animal is essential to optimise the chances of establishing a self-sustaining population. Therefore, post-release monitoring of habitat use and diet selection is necessary to inform the management of species living in their natural habitats as well as in reintroduced populations^2,3^.

Direct behavioural observations of feeding activity and analysis of faecal samples (scatology) are the two main methods that have traditionally been used to assess the dietary composition of wild animals. Behavioural observations provide direct information on food selection and foraging behaviour, whereas scatological analyses reflect the food items actually ingested and digested. However, comparative analyses of these methods are rare, and such assessments have mainly involved mammals (reviewed in^1)^. Notably, comparisons of these approaches are underrepresented in studies of birds^4^, particularly herbivorous birds. Herbivorous birds, such as large forest grouse in the family Tetraonidae, consume different plant parts throughout the year, including leaves, fruits, roots, and buds, which vary in nutritional quality and energy content^5–9^. Direct visual observation does not necessarily reveal which plant parts were ingested, and the two methods provide different levels of resolution for the taxonomic and morphological identification of plant food. Therefore, comparing the results of dietary studies derived from direct behavioural observations of food consumption (BEH) and microhistological analysis of faeces (scatology; MIC) may help to identify potential methodological biases and improve the interpretation of dietary data.

Here we explored (1) whether, and to what extent, dietary data derived from BEH and MIC studies are similar, and (2) whether they can be used interchangeably in dietary evaluations. To investigate this, we analysed BEH and MIC dietary data collected from the same population of Capercaillies *Tetrao urogallus*, a large herbivorous grouse inhabiting temperate forests. Our objectives were twofold. First, we aimed to assess the extent to which BEH and MIC dietary data are similar and correlated over the same time periods.

Second, we assessed differences in post-digestive preservation of the two most abundant plant food types (*Vaccinium myrtillus* leaves and *Pinus sylvestris* needles) to determine whether variation in tissue preservation could bias scatological estimates by over- or under-representing particular food items in faecal samples, thereby contributing to discrepancies between BEH and MIC estimates of diet composition. This is particularly important given that *V. myrtillus* is a key plant food source and a major determinant of habitat selection by Capercaillies^10^, and Scot Pine *Pinus sylvestris* is a highly preferred tree species due to its combined food and shelter availability^11–13^.

## Methods

The study was conducted in the Bory Dolnośląskie Forest (PLB020005), a continuous lowland forest area in south-west Poland covering 2,500 km² at an elevation of 140–180 m (51°21′N, 15°7′E). The material used in this study was collected as part of the Capercaillie reintroduction programme in the Bory Dolnośląskie Forest, implemented by the Ruszów Forest District since 2009.

The data used in this comparative analysis were derived from our previous studies, which employed two dietary assessment methods: direct behavioural observation of food consumption (BEH) and microhistological investigation of faeces (MIC). Detailed descriptions of both methods, along with the results of dietary studies on the studied population of Capercaillies, were presented in our two previous papers: BEH^14^ and MIC^15^.

In brief, the BEH study used a dataset combining direct observations and continuous radio tracking of 22 Capercaillies from May 2012 to April 2013^14^. We monitored the daily activity and behaviour of Capercaillies by combining radio tracking and visual observations over a period of 139 days. Each bird was monitored for an entire day once a month. A team of two observers located the roosting site of a radio-tracked bird before sunrise using radio-telemetry and collected a small subset of droppings for subsequent faecal analysis. The observers remained 10–60 m away from the bird, which allowed them to observe it with 10 × 50 binoculars throughout the day until it roosted again after sunset. The birds were usually followed continuously. However, when they flew off, the observers relocated them using radio telemetry. We recorded the time and type of behaviour (e.g., resting, feeding, moving, flying, preening), the type of plant food identified to the lowest possible taxonomic level, GPS location, and if the bird was in a tree, its height and the species of tree. During each individual foraging event, we identified the plant taxa consumed by the birds (*n* = 2,326 feeding bouts in total), but did not specify which part of the plant was consumed. Some species (e.g., two *Vaccinium* spp.) were not distinguished^14^, (Table 1). These data were then used to calculate the relative percentage of each plant taxon consumed per month or season.

**Table 1:** Comparison of two methods used to assess the dietary composition of Capercaillies: Behaviour (2,326 foraging events from 139 days of observations of 22 radio-tracked birds over one year; Merta et al., 2015) and Microhistology (a microhistological analysis of faeces (Czarnecka et al.^15^)), which expresses the percentage composition of various plant food items consumed in terms of low morpho-taxonomic division of plant taxa). A more thorough analysis of the monthly and seasonal composition of Capercaillie’s diet in the study area, based on morpho- and taxon-specific plant parts, is presented in Czarnecka et al.^15^. Food items were grouped into four major food classes: *Vaccinium* spp. (V); coniferous trees (P); monocotyledonous taxa (grass and sedge; G); and other plant foods (O).

Food item (plant taxa)	Behaviour	Microhistology
*Vaccinium* spp. (V) including *V. myrtillus* and *V. vitis-idaea*	54.47	7.18
Coniferous trees (P)		
*Pinus sylvestris*	29.58	66.90
Spruce (*Picea abies*)	2.15	0.02
Larch (*Larix decidua*)	0.13	0.10
Monocotyledonous taxa (G) (Poaceae/*Carex* spp)	10.92	14.02
Other plant food types (O)		
Heather (*Calluna vulgaris*)	1.63	-
Bracken (*Pteridium aquilinum*)	0.39	-
Meadow sweet (*Spiraea tomentosa*)	0.17	-
Wild rosemary (*Ledum palustre*)	0.17	-
Oak (*Quercus robur*)	0.09	-
Rasp- and blackberry (*Rubus idaeus*,* R. fruticosus*)	0.09	-
Birch (*Betula pendula*)	0.04	< 0.01
Beech *Fagus sylvatica*	-	1.19
Knotweed (*Polygonum* sp.)	-	0.01
Moss	-	1.50
Supplemental food†	0.17	6.07
Leaves††	-	3.03
Arthropods (A)‡	-	< 0.01
Total number of identified items	2 326	169 105

Note: different plant parts of *Vaccinium myrtillus* and *V. vitis-idaea* (*n* = 15,130 fragments) constituted 92.2% and 7.8%, respectively, of all items in the microhistological analysis of faeces. These were represented by seed fragments (66%), leaf fragments (32%), stem fragments (2%) and fruit fragments (1%).

†*Avena sativa*,* Triticum aestivum*,* Zea mays*, *Aronia melanocarpa*.

††Leaves of unidentified dicotyledonous and monocots, and other plant material.

‡Overall, 133 individuals (Araneae, ants and beetles).

Droppings were collected in the field from known Capercaillie activity sites located near night roosts. The study area covered 2,892 ha. The total number of droppings available in the field varied substantially depending on site use, ranging from a few at short-term daytime locations to several dozen at roosting sites. From this pool of collected material, 5–10 intact droppings per month were randomly selected for microhistological analysis (*n* = 80 in total). These samples were collected over consecutive months in 2013 and 2014 from sites where the presence of Capercaillies was known. Droppings were frequently collected also beneath pine trees commonly used as roosting sites by Capercaillies. Because individual identity of individuals producing the faecal samples was unknown, some samples may have originated from the same individuals. However, droppings were collected across multiple sites, and when several droppings were present at a given location, only a small subset (typically a few pellets) was collected from each accumulation to reduce the likelihood of repeated sampling of the same individuals. Given that Capercaillies in our study population travel an average of 0.9 km per day (range 0.04–3.8 km)^14^, this approach is unlikely to have resulted in substantial pseudoreplication. Thus, droppings collected within the same site and sampling occasion should be considered non-independent. Further, based on telemetry data (*n* = 3,493 GPS locations), 68.3% of movements of the Capercaillies in our study area were within 1 km and 82.5% within 1.5 km (D. Merta and J. Kobielski, unpubl. data). To minimise the likelihood of sampling the same individual, a minimum distance of approximately 1 km between sampling locations was used, corresponding to observed daily movement distances.

The MIC data were derived from microhistological analysis of the food remains found in the faeces^15,16^. A randomly selected mixed subsample of 0.1 g from each of 80 droppings was selected for detailed dietary analysis. The subsamples were divided into 1-mm² squares, with a total of 6,358 squares per Petri dish. These squares were then examined using a stereoscopic microscope at 40x magnification and the number of identifiable plant food items was counted. The samples were examined without staining, as epidermal structures of the analysed plant taxa were sufficiently distinctive for identification under stereomicroscopy. Diet composition was expressed as the number of the 49 different food items identified, including 42 types of plant material^15,16^.

Due to inconsistencies in the quality of the data between the BEH and MIC studies, we standardised both datasets by classifying the consumed food items into four major, common plant food types according to their taxonomic division: *Vaccinium* spp. (different plant parts of two species: *V. myrtillus* and *V. vitis-idaea*); needles and other parts of coniferous trees (predominantly *P. sylvestris*); monocotyledonous taxa (grasses and sedges); and other plant material (see Table 1). Due to the very low number, arthropods^15^ were excluded from the analysis. The composition of the diet per subsequent month or season was expressed as a percentage of each of the four plant food classes. We realise that this approach is a coarse generalisation of the diet, but it shows changes in the relative proportions of these food types over time in the BEH and MIC dietary assessments. Despite these data being treated as rough indices, the division into four major food types is consistent with the utilisation of these base plant taxa by Capercaillies in natural conditions and corresponds well to prior dietary data of the focal species. Most importantly, these indices are consistent with the two key plant species, *V. myrtillus* and *P. sylvestris*, which are the primary food sources for Capercaillies across their entire Eurasian range (as compiled in^8)^.

Consequently, the two methods differ in their temporal and spatial sampling frames. Behavioural observations were restricted to daylight hours, whereas faecal samples were frequently collected beneath or in close proximity to pine trees used as roosting sites. Many MIC samples originated from locations within a radius of up to 50 m from such roost trees. Thus, BEH primarily reflects daytime foraging decisions, whereas MIC integrates dietary intake over a longer temporal window that may include food ingested prior to roosting and digested overnight.

The main objective of the statistical analysis was to evaluate whether the percentages of BEH and MIC yield different estimates of dietary composition across the four main food types, and to determine which dietary components contribute most significantly to the discrepancies between the BEH and MIC data.

To evaluate the differences in multivariate diet composition between the BEH and MIC data, we used PERMANOVA (Permutational Multivariate Analysis of Variance)^17^ based on Euclidean distances calculated from square-root–transformed percentages of the four major food classes. We evaluated statistical significance using 999 random permutations to provide a nonparametric test of the null hypothesis that the multivariate centroids do not differ between BEH and MIC estimates. The proportion of variance explained by method was quantified using R². Homogeneity of multivariate dispersions was assessed using PERMDISP.

The calculations were implemented in Python using standard scientific libraries for numerical computation and permutation testing, including NumPy^18^ and SciPy^19^. Additionally, we used linear regression and Spearman’s rank correlation (rₛ) to assess the relationship between BEH and MIC percentages for the four major food types over subsequent months and seasons, using Statistica ver. 12.5^20^. The percentage data were square-root transformed to provide a more symmetric distribution and to handle zero values. Influential observations were identified using Cook’s distance (threshold = 4/n). The alpha threshold was set at 0.05.

Finally, to assess potential biases associated with varied preservation of food items, we quantified structural characteristics of epidermal fragments of *Vaccinium myrtillus* leaves and *Pinus sylvestris* needles recovered from faeces. Fragment metrics were interpreted as proxies of post-digestive preservation rather than direct measures of physiological digestibility. These metrics were not used to adjust microhistological counts or to estimate detection probabilities. This approach allowed us to assess whether differences between the BEH and MIC estimates for the two dominant food types (i.e., *Vaccinium* plant parts and *P. sylvestris* needles) could be partly explained by tissue-specific preservation biases inherent to microhistological analysis (sensu^21)^. Measurements were taken during microscopic examination. Using CellSense Dimension ver. 1.5^22^, we quantified the surface area (mm²) and perimeter (mm) of individual epidermal particles of *P. sylvestris* needles and *V. myrtillus* leaves. Additionally, the number of 1-mm² grid squares covered by each particle was recorded. Differences between particle types were tested using one-way ANOVA.

## Results

Both behavioral observation (BEH) and scatology (MIC) metrics indicated that the three major food items were *Vaccinium* (different plant parts), coniferous trees (mostly conifer needle remains, predominantly from *P. sylvestris*), and monocotyledonous taxa, which accounted for 95% and 88% of all identified items in the BEH and MIC data sets, respectively (Table 1).

**Fig. 1:**
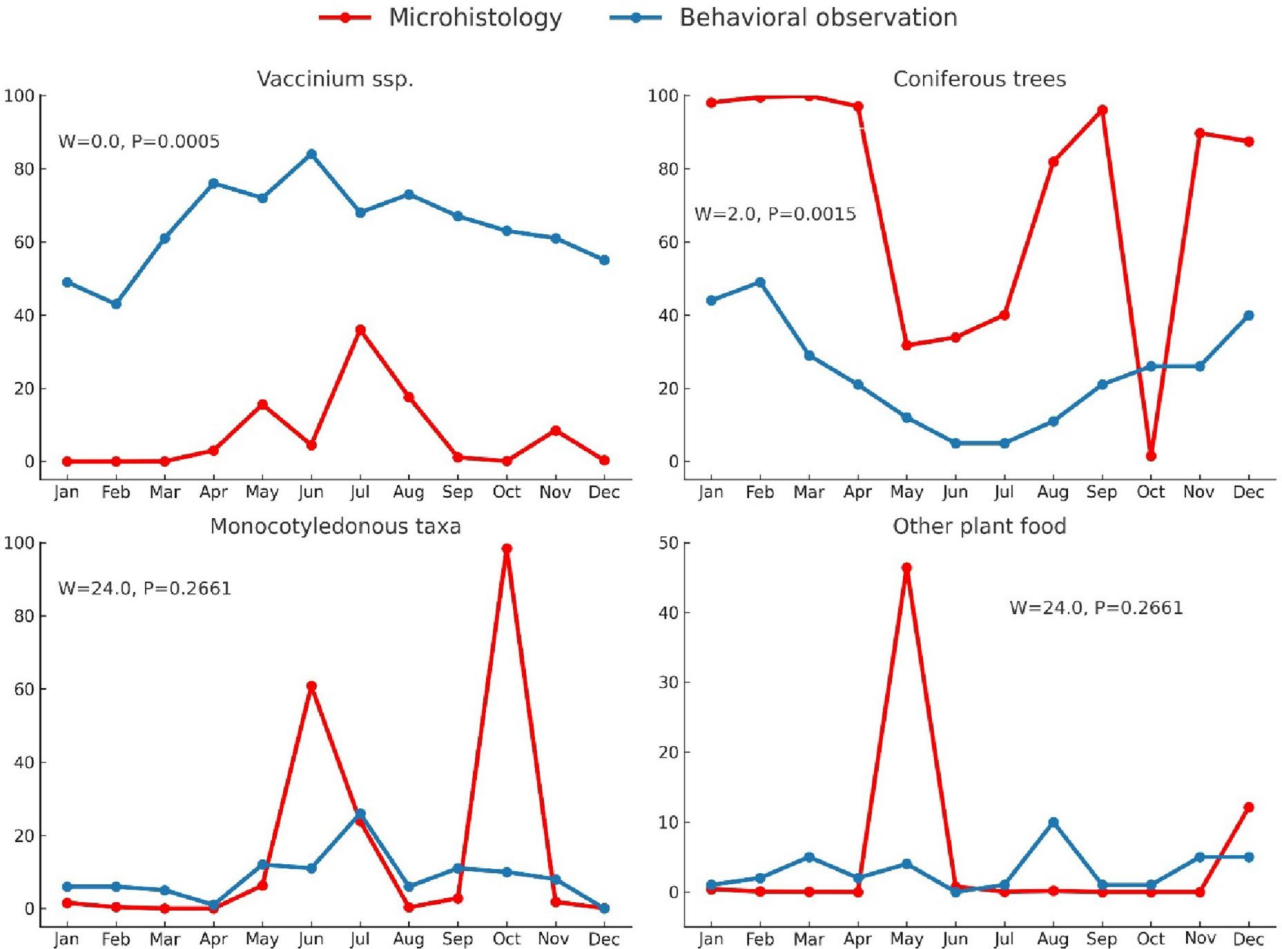
Comparison of the monthly percentages (y-axis) of the four main plant food categories consumed by Capercaillies, as determined by microhistology (MIC) and behavioral observation (BEH). The values represent Wilcoxon signed-rank test statistics (W) and the associated p-values, which assess paired differences between the percentages obtained from the two approaches.

PERMANOVA revealed a highly significant difference in the composition of the four major food types between the BEH and MIC estimates (pseudo-F₁,₂₂ = 42.09, *P* = 0.001), with method explaining 51.3% of total variance in dietary composition (R² = 0.513). However, PERMDISP indicated significant differences in multivariate dispersion between methods (F₁,₂₂ = 11.53, *P* = 0.003), suggesting that part of the observed separation may be attributable to differences in within-group variability. Together, these results demonstrate that the primary multivariate separation identified by PERMANOVA was mainly driven by differences in the proportions of *Vaccinium* and conifer needles, as determined by Wilcoxon tests (Fig. 1).

**Fig. 2:**
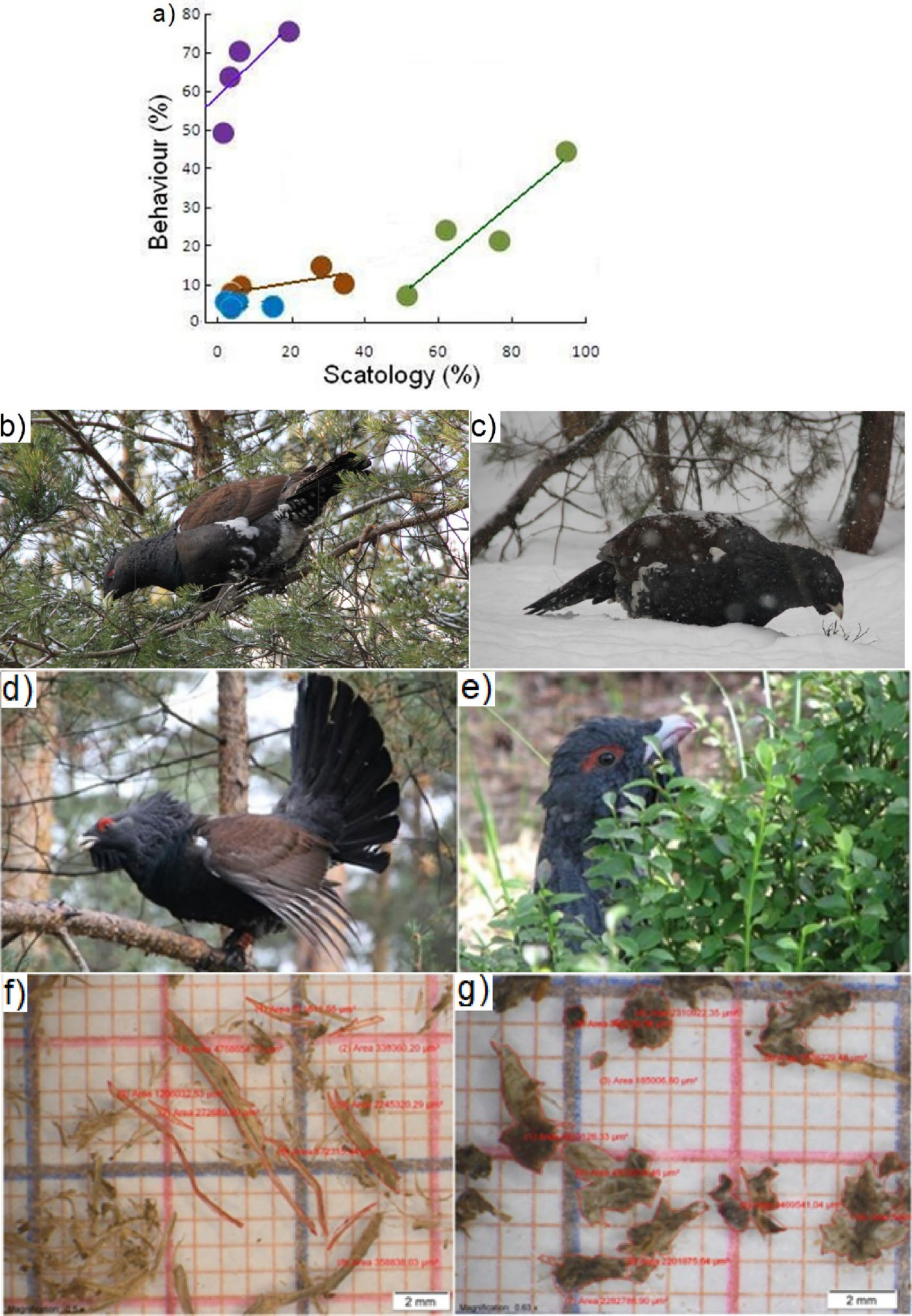
Relationships (**a**) between estimates of the four main plant food categories consumed in the four seasons: spring (March–May), summer (June–August), autumn (September–November) and winter (December–February), derived from behavioural observations (BEH, y axis) or scatology (MIC, x axis). Differently coloured points represent the average values determined for various parts of *Vaccinium* spp. (purple), coniferous tree needles (mostly *Pinus sylvestris*; green), monocotyledonous taxa (grasses and sedges, including cereals; brown), and other plant food types (mosses, deciduous trees, and other plant material; blue) in the Cappercaillie diet. (**b**) A cock feeding on pine needles.(**c**) feeding on shoots of *Vaccinium myrtillus* protruding above the snow. (**d**) A cock roosting on a pine tree (**c**) ingesting fruits of *V. myrtillus*, as evidenced by the purple coating on the beak (© Janusz Kobielski). Differences in post-digestive preservation of plant tissues in faeces observed under a microscope at 30× magnification on 1-mm² grid squares: *Pinus sylvestris* needle fragments (**f**) and *Vaccinium myrtillus* leaf fragments. (**g**) Fragment surface area (mm²) is indicated in red. *Note*: Seasonal relationships are based on four seasonal averages, and none of the presented relationships were statistically significant (*P* ≥ 0.233). For monthly relationships (*n* = 12), see Supplemental Fig. S1.

Seasonal comparisons did not reveal significant relationships between BEH and MIC percentages of the four major plant food types (Fig. 2a).

Monthly correlations between BEH and MIC estimates (Supplemental Fig. S1) based on square-root–transformed percentage data showed limited linear relationships (*Vaccinium*: R² = 0.30, *P* = 0.063; conifer needles: R² = 0.18, *P* = 0.173; monocotyledonous taxa: R² = 0.28, *P* = 0.075). After removing an influential observation for conifer needles (identified using Cook’s distance), the relationship became significant (R² = 0.548, *P* = 0.009). Spearman’s rank correlations, however, indicated broadly consistent month-to-month variation between methods for *Vaccinium* (rₛ = 0.71, *P* = 0.010), conifer needles (rₛ = 0.63, *P* = 0.027) and monocotyledonous taxa (rₛ = 0.87, *P* < 0.001), despite differences in absolute percentages.

More specifically, the mean monthly proportion of *Vaccinium* spp. plant parts was nine times lower in the MIC study than the BEH values (7% vs. 64%, respectively). During five months (January, February, March, September and October), the percentage of *Vaccinium* spp. in the MIC study remained ≤ 1.15%, and it was completely absent in December (Fig. 1). Conversely, the percentage of conifer needles was three times higher in the MIC study than in the BEH study (71% vs. 24%, respectively) (Fig. 1, Supplemental Fig. 2). The MIC and BEH percentages of monocotyledonous taxa show strong dissimilarities in June and October (Fig. 1), resulting from a higher contribution of sedges (*Carex* spp.) in June and cereals in October in the faecal samples^15^. The MIC and BEH percentages of other plant food types differed mainly in May, likely due to the high contribution of mosses and beech buds (*Fagus sylvatica*) found in the faecal samples in this month (Table 1).

Notably, the plant parts of *Vaccinium myrtillus* and *Vaccinium vitis-idaea* (15,130 fragments identified in total) constituted 92.2% and 7.8%, respectively, of all items in the examined faecal samples. The majority of these were fragments of seeds (66%), followed by remains of leaves (32%), stems (2%) and fruits (1%). Furthermore, 917 and 53 intact seeds of *V. myrtillus* and *V. vitis-idaea*, respectively, were recovered from the examined faecal samples (Czarnecka et al.,^15^).

### Differential preservation of plant fragments in faeces

Visual inspection of *V. myrtillus* leaf and *P. sylvestris* needle remains showed an apparent difference in fragment morphology and post-digestive preservation. Specifically, the needle remains were narrow and elongated, and contained well-preserved stomata (Fig. 2f), whereas the *V. myrtillus* leaf remains were polygonal (Fig. 1g). The average fragment size of *V. myrtillus* leaf remains in faecal material was nearly twice that of needle remains (2.67 ± 0.53 mm² vs. 1.40 ± 0.52 mm², respectively), and this difference was highly significant (one-way ANOVA, F = 11.59, *P* < 0.001).

Further, the average perimeter of needle fragments was 17% larger than that of *V. myrtillus* leaf fragments (10,009 ± 2,108 μm vs. 8,530 ± 1,187 μm, respectively). Finally, needle fragments covered 9% more 1-mm² grid squares than *V. myrtillus* leaf fragments (5.66 ± 1.24 vs. 5.21 ± 0.86, respectively).

## Discussion

Our comparative evaluation of two widely used dietary assessment methods shows that behavioural observations (BEH) and microhistological analysis of faeces (MIC) provide complementary but method-dependent perspectives on the year-round diet of Capercaillie, and that they differ not only in estimated diet composition but also in the temporal variability of dietary estimates. The absolute proportions of major food classes differed substantially between methods. The estimated contribution of *Vaccinium* plant parts was approximately nine times lower in microhistological analysis than in behavioural observations (7% vs. 64%), whereas the contribution of conifer needles was about three times higher in MIC (71% vs. 24%) than in BEH. Despite these differences, both approaches identified *Vaccinium* spp. and conifer needles (predominantly *Pinus sylvestris*) as dominant components of the diet, and rank correlations indicated broadly similar month-to-month variation between methods. Importantly, BEH and MIC should not be interpreted as interchangeable estimates of diet composition but rather as complementary indicators reflecting different temporal and behavioural aspects of food intake.

The comparison of dietary data derived from BEH and MIC highlights a common challenge in dietary analysis: discrepancies between an animal’s apparent diet based on behaviour and the diet reconstructed from digested remains. In our study, these differences reflect a combination of differential digestibility, diel feeding patterns and inherent methodological constraints. The observed divergence in proportional estimates is best explained by differences in temporal integration and sampling context rather than methodological inaccuracy. Behavioural observations capture daytime foraging decisions, whereas faecal samples integrate consumption over longer periods, including feeding associated with roosting sites. Notably, MIC data indicate a strong dominance of conifer needles during the autumn-winter period, whereas behavioural observations suggest a greater contribution of *Vaccinium* plant parts. This discrepancy likely arises from the combined effects of diel feeding behaviour and differential post-digestive representation of plant tissues.

Capercaillies frequently roost in conifer trees^12,23,24^, and individuals in our study population showed the same pattern, consistently using trees as roost sites (Fig. 1d). Previous studies further indicate that conifer needles may be ingested preferentially in late afternoon and digested overnight^11,25^. During our field observations, we recorded that Capercaillies typically foraged intensively in the ground vegetation layer shortly before flying to roosting sites. Feeding on pine needles was observed mainly after birds had settled in their roosting trees. This pattern suggests that a substantial proportion of conifer intake may occur during the roosting period rather than immediately beforehand. However, under conditions of deep snow cover, which limits access to ground vegetation, birds were frequently observed spending extended periods in trees throughout the day (Fig. 1b-e), moving between them and feeding on pine needles continuously. In such conditions, conifer needles may constitute the dominant food source over the entire daily activity period. Our observations also indicate that individuals typically remained close to their roosting trees after sunrise, usually within approximately 50 m (Merta D., Kobielski J. – unpubl.). Consequently, even when droppings were collected away from roosting trees, their composition may still largely reflect food items ingested earlier, particularly food types that are structurally resistant to digestion such as pine needles. Because BEH observations were limited to daylight hours, feeding associated with roosting behaviour may therefore have been underrepresented in behavioural records.

Differential preservation of plant tissues during digestion may additionally contribute to discrepancies between methods. *Vaccinium* leaves and soft tissues are likely to fragment extensively and may be less readily identifiable in faecal material, whereas conifer needles possess structurally resilient epidermal tissues that remain recognisable after passage through the digestive tract^21,26,27^. Our fragment measurements suggest differences in post-digestive preservation. However, these metrics do not directly quantify digestibility or nutrient assimilation. Therefore, preservation bias alone is unlikely to fully explain the magnitude of divergence observed between BEH and MIC estimates. Similar methodological biases have been noted in earlier studies of Capercaillie diet. Based on a comparative analysis of behavioural observations and analyses of digestive tract contents and faeces, Semenov-Tyan-Shansky (1960)^26^ showed that faecal analyses tend to overestimate poorly digestible components such as conifer needles and woody shoots, while easily digestible food items (leaves) may remain largely undetected. Consistent with this interpretation, the higher digestibility of *Vaccinium* leaves relative to conifer needles likely results in underrepresentation of *Vaccinium* tissues and overrepresentation of needles in faecal samples^21^, although this mechanism alone cannot fully explain the magnitude of the discrepancy observed between methods.

We found strong mismatches between the percentage of *Vaccinium* plant parts, coniferous trees, monocotyledonous taxa and other plant material in BEH and MIC, as these indices were not significantly intercorrelated over subsequent months.

Specifically, the MIC data indicated that Capercaillies feed mostly or entirely on pine needles, whereas BEH data indicated that *Vaccinium* plant parts dominated the diet.

Furthermore, the disproportionately higher proportion of *Vaccinium* seed fragments (66%) compared to leaves (32%) in faecal samples may reflect a combination of differential digestion of plant tissues and selective consumption of berries over leaves, thereby being consistent with frugivory (see Fig. 1e). As behavioural observations did not allow reliable differentiation between specific plant parts consumed, it remains unclear to what extent these patterns result from digestive processes versus feeding selectivity. Comparable patterns may also arise in the absence of leaf consumption, due to the differential detectability of seeds and leaf tissues in faecal material. The dominant mismatch between BEH and MIC involved conifer needles, a food type not affected by the aggregation of different *Vaccinium* plant parts, indicating that the discrepancy is unlikely to result solely from differences in food classification. In addition, the relatively small number of droppings analysed in some months (*n* = 5) may reduce precision in monthly diet estimates. The relatively high proportion of conifer needles detected in the MIC dataset is consistent with the late-afternoon feeding and prolonged digestion of needles associated with roosting. This interpretation is consistent with previous findings showing that foraging and roosting can occur in the same tree^12,28–31^. It is also supported by observations that Capercaillies can fill their crops with needles once per day and digest these food items over extended periods^11,25^. Furthermore, De Franceschi and Boag^32^ documented diel differences in the consumption of *Vaccinium* leaves and conifer needles in Spruce Grouse *Dendragopus canadensis*, with conifer needles consumed more intensively later in the day. However, the potential shift in the Capercaillie’s diet between nocturnal roosting sites and daytime foraging areas has not yet been investigated and warrants further study.

Conversely, behavioural observations may also overestimate the use of low understorey vegetation such as *Vaccinium* while underrepresenting feeding occurring in the tree canopy (Fig. 1b). This pattern may be particularly pronounced in winter, when *Vaccinium* shoots can remain accessible above the snow layer (see Fig. 1c) and can constitute an important component of the diet in behavioural observations. Winter weather conditions may further influence this pattern, as deeper snow cover limits access to ground vegetation and increases reliance on conifer needles^33^.

Both MIC and BEH approaches therefore provide complementary but method-specific perspectives on diet composition. Faecal analyses are non-invasive, allow large sample sizes and provide detailed identification of plant remains. However, differential digestibility can bias representation of food types. Behavioural observations provide valuable information on plant selection, foraging context and habitat use, but are limited by visibility constraints and restriction to daylight observations.

Finally, despite the contrasting perspectives provided by MIC and BEH approaches, a comprehensive understanding of the dietary ecology of galliform birds requires integrating insights from both methods. Behavioural observations provide information on plant selection and habitat context, whereas scatological analyses allow identifying food items at a high morpho-taxonomic level and the potential for seed dispersal and predation. Researchers should therefore consider potential methodological biases, including those associated with the timing of sampling^32,34^. Future studies using infrared wildlife cameras could help document poorly known crepuscular and nocturnal activity of Capercaillie and other forest birds^34–36^, which may further clarify the mechanisms underlying discrepancies between dietary assessment methods.

## Data Availability

The datasets analysed during the current study are available in the Zenodo repository 10.5281/zenodo.15510661 and described in our companion data article published in *Scientific Data* 10.1038/s41597-025-05811-1.
